# Salivary Antioxidant Status in Patients with Oral Lichen Planus: Correlation with Clinical Signs and Evolution during Treatment with* Chamaemelum nobile*

**DOI:** 10.1155/2018/5187549

**Published:** 2018-05-27

**Authors:** Asta Tvarijonaviciute, Cristina Aznar-Cayuela, Camila P. Rubio, Fernando Tecles, Jose J. Ceron, Pia López-Jornet

**Affiliations:** ^1^Interdisciplinary Laboratory of Clinical Analysis Interlab-UMU, Regional Campus of International Excellence “Campus Mare Nostrum”, University of Murcia, 30100 Espinardo, Murcia, Spain; ^2^Department of Oral Medicine, Faculty of Medicine, University of Murcia, 30100 Espinardo, Spain

## Abstract

Oral lichen planus (OLP) is a chronic inflammatory mucocutaneous disease, which manifests as a succession of outbreaks. OLP was associated with salivary oxidative stress. Randomized, double blind, parallel-group study was performed. The sample consisted of 55 clinically and histopathologically diagnosed OLP patients. Twenty-six patients were treated with 2%* Chamaemelum nobile* gel and 29 with a placebo. Nonstimulated (basal) saliva was collected on the first day of the study and 4 weeks later. Salivary total antioxidant status (TAS) was evaluated by four different methods: two TAC (6-hydroxy-2,5,7,8-tetramethylchroman-2-carboxylic acid) equivalent antioxidant capacity methods (TAC1 and TAC2), cupric reducing antioxidant capacity (CUPRAC), and ferric reducing ability of plasma (FRAP). At baseline (T1), no statistically significant differences were detected in any of the TAS analytes between the two groups of patients. After four weeks of treatment, a statistically significant increase was detected in FRAP in the placebo group (0.323 [0.090–0.467] versus 0.406 [0.197–0.848] mmol/g⁎10^−3^) (*P* < 0.05). Significant correlations were observed between pain and drainage and TAC1, CUPRAC, and FRAP and between xerostomia and the TAC1, TAC2, CUPRAC, and FRAP. The results of the present study showed that in patients with OLP increases of TAS in saliva are associated with increase in pain and xerostomia and decrease in drainage, suggesting a worsening condition of the patient. The use of* Chamaemelum nobile* gel would be recommended for disease stabilization.

## 1. Introduction

Oral lichen planus (OLP) is a chronic inflammatory mucocutaneous disease. OLP manifests as a succession of outbreaks that can adopt a variety of clinical forms: reticular OLP with Wickham's Striae, erosive-ulcerous OLP, and atrophic OLP. Its high prevalence (0.5–2%), recurrent nature, and potential risk of malignancy have led to extensive research into the disease [1, 2].

The etiology of OLP is unknown, although its pathogenesis includes an immune disorder in which CD8 cytotoxic lymphocytes attack epithelial cells [2, 3]. There is a hypothesis that increased oxidative stress and an imbalance in the antioxidant defense system may be involved in the pathogenesis of OLP [4, 5]. For this reason, it is thought that determining the oxidative/antioxidant status of an inflammatory disease may be of value for assessing its severity and for monitoring the disease's evolution and response to treatment [6, 7].

In order to clarify the possible association between the oxidative stress and OLP pathogenesis, measurement of antioxidants and oxidants in saliva of patients suffering from this disease is increasing in the last years [8]. Saliva offers several advantages over serum as a diagnostic fluid: its collection is noninvasive and very easy, and sample collection can be repeated indefinitely. Recently, alterations in total antioxidant status (TAS) and reactive oxygen species (ROS) were described in saliva of patients with OLP when compared with healthy controls [8]. However, knowledge about the behavior of analytes related to oxidative stress in saliva is lacking [7].

The most common therapeutic options for treating OLP include corticosteroids, retinoids, cyclosporine, tacrolimus, phototherapy, and surgery, although treatment often produces adverse effects [2, 3]. Chamomile has been used to deal with diverse inflammatory disorders and possesses a variety of active flavonoids such as alpha bisabolol, azulene, matricin, and chamazulene, all of which have antioxidant, anti-inflammatory, antispasmodic, antibacterial, and immunoregulatory capacities [9]. For this, chamomile was shown to be beneficial in the treatment of oral diseases, such as mucositis [10]. Furthermore, the topic chamomile application was shown to improve clinical presentation of OLP, including decreased pain, burning sensation, and itching [11].

This study investigated the correlation of TAS of saliva in patients with OLP measured by four different assays and its possible relation to clinical variables such as pain, draining, and xerostomy. Furthermore, the changes of TAS during OLP treatment were assessed.

## 2. Material and Methods

### 2.1. Study Design

This randomized, double blind, parallel-group study, of 4-week duration (Trial Registration Number Identifier: NCT02421770) was conducted in full accordance with ethical principles and was approved by the Bioethics Commission of the University of Murcia. Informed consent to take part was obtained from each subject.

Saliva samples were collected from patients diagnosed with OLP following established clinical and histologic criteria [6]. Included patients had not received any treatment for OLP in the previous two weeks in the case of topical treatments, or in the last four weeks in the case of systemic therapies. Exclusion criteria consisted of allergy to some ingredient of the products tested, the use of antioxidant drugs or medication capable of inducing lichenoid reactions, the presence of dysplasia in the histopathological study of OLP, periodontal disease, medication with an immunosuppressant, and a history of trauma and/or surgery.

### 2.2. Study Products

The product assayed was 2%* Chamaemelum nobile*, with a gel consistency supplied in 500 ml containers, as was the placebo [11]. Both products consisted of the same excipients and composition, water, hydroxyethyl, sorbitol < 0.1%, E-202 (potassium sorbate) < 0.1%, E-223 (sodium metabisulfite) < 0.1%, food coloring < 0.1%, and chamomile aroma < 0.1% (Ababbo, Murcia, Spain), except that the experimental gel included 2% chamomile and the placebo did not. Both preparations had the same colour. An operator external to the study coded the products in identical opaque containers. A randomization code was kept in an opaque envelope in a safe environment and opened only at the end of the study. Both patients and researchers were blind to group assignment (treatment/placebo). The gels (0.5 ml) were applied uniformly to the oral cavity with the finger three times a day in the areas that presented symptoms for a period of 4 weeks. After each application, patients were asked not to eat or drink for 20 min.

A clinical history was made for each patient and patients were examined clinically. Oral clinical examinations and data registration were performed by a single examiner, a specialist in oral medicine (CA). Patients were asked to indicate their estimated mean pain intensity at the beginning and the end of the trial. The pain was measured on a 10-point Visual Analogue Scale (VAS) (0 = no symptoms, 10 = severe pain) (López-Jornet et al., 2009). The patients were asked to draw a vertical line at the point on the horizontal line which best represented their symptoms. Draining was evaluated as previously described and salivary flow rates were measured in ml/min [12]. The xerostomy inventory was evaluated using a questionnaire consisting of 11 items. Patients respond by scoring from zero to five according to the absence (0) or severity (5) of the symptom, a higher score indicating greater severity [13].

### 2.3. Saliva Collection

Before collecting saliva, all participants rinsed their mouth with distilled water. Nonstimulated saliva was obtained using the draining method [12], without chewing movements, in dry plastic vials with the participant sitting in a relaxed position during 5 min. In all cases, saliva samples were taken in the morning between 10.00 and 12.00 hours. Samples with blood contamination (determined by visual inspection scale) were excluded, since unvisible blood contamination of saliva does not interfere with oxidative stress markers and antioxidant status [14]. Saliva was centrifuged immediately after collection at 3000*g* for 10 min. The supernatant was transferred into Eppendorf tubes and stored at −80°C until analyses.

### 2.4. Antioxidant Analysis

Salivary TAS was evaluated by measuring trolox equivalent antioxidant capacity (TAC1 and TAC2), ferric reducing ability of plasma (FRAP), and cupric reducing antioxidant capacity (CUPRAC) as previously described [8].

### 2.5. Statistical Analysis

All data were registered as medians and percentiles (unless otherwise stated). These were calculated using routine descriptive statistical procedures and software (GraphPad Software, San Diego, CA, USA). Results were evaluated for approximate normality of distribution using the D'Agostino and Pearson omnibus normality test, giving a nonparametric distribution; for T1 and T2 comparison within the groups, and to make comparisons between groups, data were log transformed and Student's *t*-test was used. Correlations between variables were estimated using Spearman correlation analysis. A value of *P* < 0.05 was considered statistically significant.

## 3. Results

**Figure 1:**
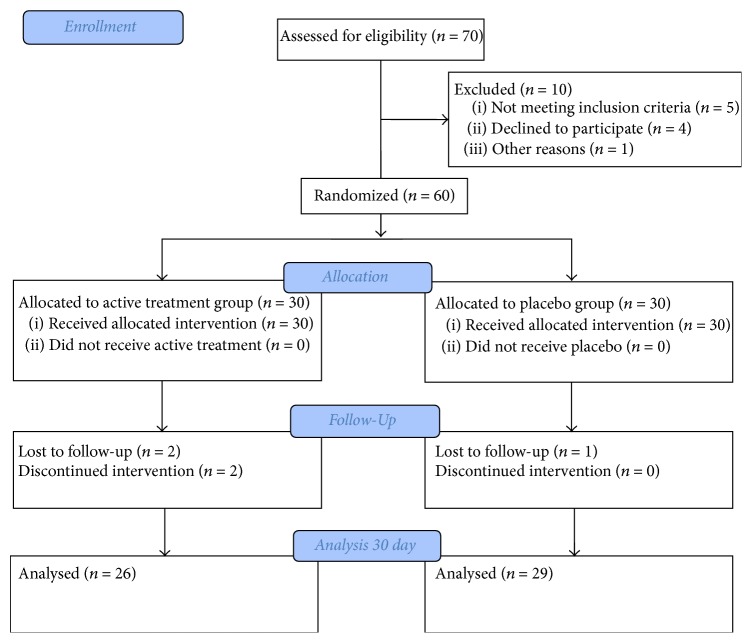
Flow diagram.

Finally, saliva samples before and four weeks after the treatment were obtained from a total of 55 patients (Figure 1). In the treatment group, 10 subjects were male (38.5%) and 16 were female (61.5%); in the placebo group, 7 (24.1%) were male and 22 (77.9%) female (*P* = 0.25). Mean age in the treatment group was 63.1 ± 14.36 years and 62.8 ± 10.3 in the placebo group (*P* = 0.91).

No statistically significant differences were found in either group with respect to OLP evolution time, which ranged from 6 months to 8 years. Median (range) OLP severity was 2 (2–4) in the treatment group and 2 (2–4) in the placebo group (*P* = 0.58)

Table 1 shows median (interquartile range) data of TAC1, TAC2, CUPRAC, and FRAP at baseline (T1) and at four weeks for both groups. At baseline (T1), no statistically significant differences were detected in any of the analytes between the two groups of patients. After four weeks of treatment, a statistically significant increase was detected in FRAP in the placebo group (*P* < 0.05). No other statistically significant changes were detected for any of the other analytes.

When data of the two groups and the two samplings were pooled, significant correlations were observed between pain and drainage and TAC1, CUPRAC, and FRAP, and between xerostomia and the TAC1, TAC2, CUPRAC, and FRAP (Table 2).

## 4. Discussion

There are evidences that OLP is associated with oxidative stress in saliva since various oxidative biomarkers such as TAS, malondialdehyde (MDA), uric acid, or gamma-glutamyl transferase [4, 8, 15–17] were shown to be altered in this disease. Salivary TAS was previously measured in healthy controls and patients with OLP [4, 8, 18]. However, no studies were reported about the dynamics of TAS in saliva of patients with OLP after treatment as well as the possible correlations between TAS values and severity of OLP clinical signs.

Salivary antioxidant capacity can be measured with a variety of methods. The present study employed TAC1, TAC2, CUPRAC, and FRAP, which are the most widely used methods for measuring total antioxidant status. Furthermore, it was suggested that the best approach was to combine different assays when evaluating TAS [8, 19]. Determination of antioxidant status in a global way by TAS assays offers the benefit of evaluation of all individual antioxidant components of a sample, in contrast to determination of each antioxidant component separately, what is labor-intensive, time-consuming, and costly-expensive [20, 21]. However, different assays employed for TAS determination could produce diverse results and conclusions, since they measure different compounds. For instance, measurement of trolox equivalent antioxidant capacity (TAC) reflects plasma concentrations of albumin, urate, ascorbic acid, a-tocopherol, and bilirubin, while FRAP values mainly reflect levels of uric acid (up to 60%) and less ascorbic acid and a-tocopherol [21, 22]. For this reason, in the present study, salivary TAS was evaluated by four different methods. Although it should be stated that none of these methods are able to measure enzymes [23]. Therefore, the activity of some enzymatic oxidative stress markers, such as superoxide dismutase, catalase, or glutathione peroxidase, is not reflected in TAS measurements [23].

Patients included in the present study presented a mild to moderate form of the disease (median severity index was 2 out of 5 in both groups), what allowed the application of alternative treatment or including them in placebo group. Otherwise, it would be ethically not appropriate to include severely ill patients and maintain them during one month without any treatment (in case of the placebo group). This fact should be taken in account when extrapolating the results to other studies, since different severity of the disease could result in different behavior of antioxidants [24].

The increase in FRAP that occurred in the placebo group would indicate that an increase in TAS occurs when patients with OPL are not treated. Although unfortunately no markers of oxidative stress were measured in our study, it could be postulated that the increase in TAS in nontreated patients with median-low severity OLP would be related to an increase in the need of antioxidant protection secondary to an increase in oxidant compounds in saliva. Elevated concentrations of oxidant compounds in saliva were associated with lichen planus and correlated with the severity of the lesions [5]. In a previous study, we have demonstrated that the TAS concentrations in saliva are higher in patients with OLP compared to controls. And in line with our study, Agha-Hosseini et al. [18] found increased salivary TAS, using FRAP method, in patients with OLP in comparison with controls, although this was not statistically significant. In contrast, Ergun et al. [4] found no significant differences in salivary FRAP between healthy and OLP patients. This disagreement at least in part could be explained by the inclusion of patients with different severity OLP, although further studies would be indicated in order to clarify this topic.

The samples used in this study were banked samples of a previous report in which the use of chamomile topical treatment for one month was associated with the clinical improvement in patients with OLP [11]. Despite the clinical improvement, we did not detect changes in salivary TAS. However, the lack of increase of TAS in treated group could be considered as a beneficial effect of the chamomile resulting in disease stabilization and, thus, its use could be recommended for patients with OLP severity score of 2–4 out of 5. Further long-lasting studies using higher % preparations should be desirable in order to evaluate the possible effects of chamomile on salivary markers related to inflammation and oxidative stress in patients with OLP.

When clinical variables were evaluated in relation to salivary TAS levels, positive correlation was observed between TAS and pain, being the strongest when FRAP assay was used. This finding at least in part could be explained by the fact that pain results in increased cortisol (an endogenous glucocorticoid), which in turn was associated with increased antioxidant capacity of the serum [25, 26]. Furthermore, negative and positive correlations between salivary TAS and drainage and xerostomy, respectively, were observed. These two correlations complement and confirm one another and indicate that in cases where less saliva is produced and thus the feeling of dry mouth is increased, salivary TAS levels are higher.

In conclusion, the results of the present study showed that, in patients with OLP, the topical application of chamomile for one month had no effect on salivary TAS, while the lack of treatment (placebo group) was accompanied by the significant increase in antioxidants measured by FRAP assay. Furthermore, an increase in salivary antioxidants in patients with OLP, especially when measured by FRAP method, was related to an increased pain and xerostomy and a decreased drainage. Taken together, it could be suggested to use 2% chamaemelum nobile gel to stabilize patients with median-low severity OLP.

## Figures and Tables

**Table 1:** Median (interquartile range) data for TAC1, TAC2, CUPRAC, and FRAP at baseline (T1) and four weeks after (T2) in placebo and treatment groups.

Analyte	Placebo			Treatment		
	T1	T2	*P*	T1	T2	*P*
Flow rate, ml/min	4.7 (1.4–7.8)	4.1 (2.0–7.5)	0,427	3.6 (1.5–9.0)	5.1 (1.2–7.5)	0.4672
Xerostomy	19 (11–30)	22 (13–33)	0.457	24 (12–34)	22 (12–31)	**0.048**
TAC1, mmol/L	0,201 (0,136–0,299)	0,233 (0,150–0,393)	0,390	0,288 (0,166–0,404)	0,278 (0,209–0,445)	0,477
TAC2, mmol/L	0,400 (0,258–0,589)	0,421 (0,223–0,522)	0,972	0,457 (0,295–0,513)	0,397 (0,276–0,526)	0,946
CUPRAC, mmol/L	0,130 (0,088–0,255)	0,191 (0,116–0,359)	0,401	0,199 (0,113–0,348)	0,225 (0,149–0,408)	0,391
FRAP, mmol/L	0,323 (0,090–0,467)	0,406 (0,197–0,848)	**0,039**	0,392 (0,195–0,685)	0,464 (0,298–0,568)	0,781

**Table 2:** Correlation between total antioxidant capacity and clinical parameters.

Variable	Pain	Flow rate	Xerostomy
	*r*; *P*	*r*; *P*	*r*; *P*
TAC1, mmol/L	NS	NS	0.233; 0.013
TAC2, mmol/L	0.239; 0.010	−0.323; 0.001	0.326; 0.001
CUPRAC, mmol/L	0.182; 0.040	−0.359; <0.001	0.271; 0.005
FRAP, mmol/L	0.331; <0.001	−0.345; <0.001	0.322; 0.001

## Abbreviations

CUPRAC: Cupric reducing antioxidant capacity
FRAP: Ferric reducing ability of plasma
OLP: Oral lichen planus
ROS: Reactive oxygen species
TAS: Total antioxidant status
TAC: Trolox (6-hydroxy-2,5,7,8-tetramethylchroman-2-carboxylic acid) equivalent antioxidant capacity method.
